# Living histopathology - interrogation of ocular tissues by light: a celebration of the slit-lamp and a repertoire of clinical techniques

**DOI:** 10.1038/s41433-024-03139-5

**Published:** 2024-10-16

**Authors:** Paul A. R. Meyer

**Affiliations:** Visiting Fellow, Cambridge University Department of Engineering; Hon Senior Visiting Fellow, Cambridge University Department of Medicine; Retired Consultant Medical Ophthalmologist, Addenbrooke’s Hospital, Cambridge, UK

**Keywords:** Eye manifestations, Biotechnology

## Abstract

The evolution of the slit-lamp microscope has enabled ophthalmologists to examine the transparent tissues of the eye with histological detail. This paper considers the history and optics of the slit-lamp. Optical sectioning and retro-illumination are discussed; particularly, effective placement of the reflected light beam. A variety of less conventional slit-lamp examination techniques is described. These include remote dark-field retro-illumination, examination through refractive surfaces (particularly, meniscus retro-illumination to demonstrate tear cells and non-contact corneal endothelial specular microscopy), location of vitreous abnormalities by parallax, expanding radial cords of vitreous cells in lymphoma, mirror examination of the superior fornix and corneal epithelial folds in ocular hypotension. It concludes with brief discussions about haemoglobin video imaging, semi-quantification of aqueous outflow volume by aqueous column cross-section area, and autofocus for video-microscopy.

## Abstract (zh)

裂隙灯显微镜的发展使眼科医生能够检查眼部透明组织的组织学细节。本文阐述了裂隙灯的历史和光学原理, 讨论了光学切片和反向照明, 尤其是反射光束的有效设置。本文描述了多种非常规裂隙灯检查技术。其中包括远程暗视野逆行照射、透过屈光面进行检查(尤其是凹凸透镜逆行照射以显示泪液细胞和非接触式角膜内皮镜检查)、通过视差定位玻璃体异常、淋巴瘤患者玻璃体细胞放射束扩张、低眼压时上穹隆和角膜上皮的皱褶。本文简要讨论了血红蛋白视频成像、利用水柱横截面积半定量测定房水流出量, 以及视频显微镜自动调焦。

## Introduction

The evolution of the slit-lamp during the first half of the 20th Century was arguably the most significant development in the examination of living subjects since the art of physical examination.

The corneal biomicroscope, developed in 1887 by **Wilhelm von Zehender** and **Heinrich Westein** [1], established binocular microscopy of the anterior segment of the eye; however, innovative illumination was needed before the instrument could realise its full potential. Over time, it would enable the anatomy and pathology of transparent ocular tissues to be examined with single cell resolution, in vivo.

**Fig. 1:**
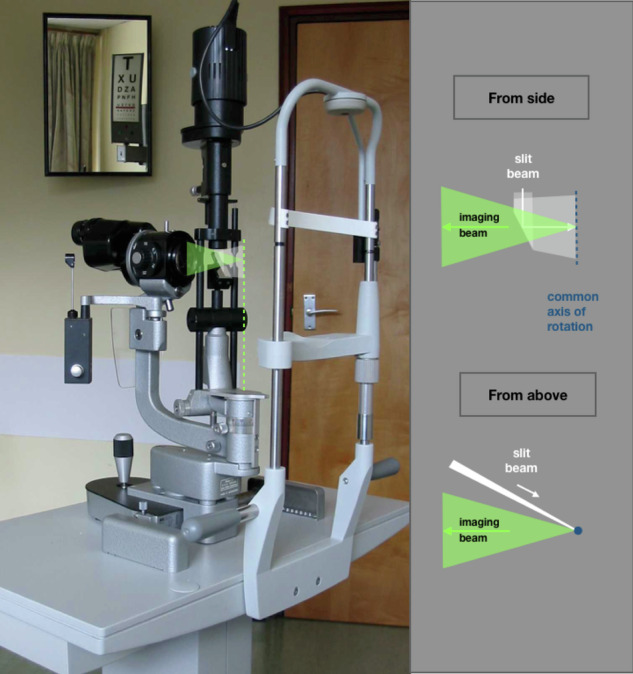
Slit-lamp.

In the modern slit-lamp, the eye is illuminated by a collimated ribbon of light (the slit beam) and examined with a long working-distance, binocular, compound microscope. The slit light source and microscope are mounted on two independent brackets, which share a common axis of rotation, centred on the tissue under examination. However, the slit can also be re-directed to dissociate illumination from the microscope field (Fig. 1).

## Evolution of the slit-lamp microscope

**Fig. 2:**
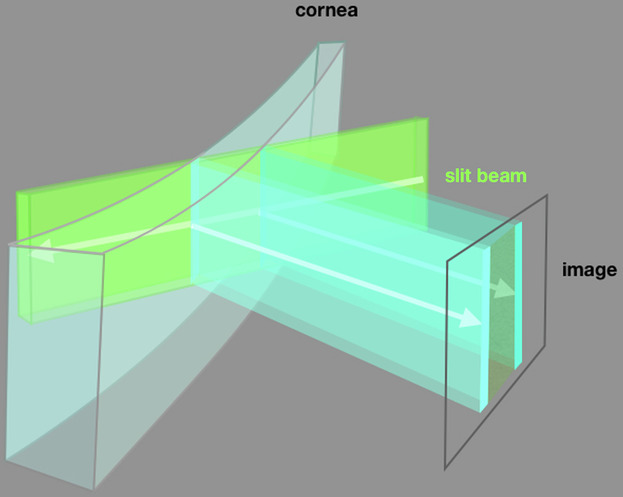
Slit illumination. As a ribbon of light passes through a transparent tissue, it is scattered by each interface at which refractive index changes. When the illumination and imaging axes intersect at an acute angle, back-scatter demonstrates the interfaces, separated spatially in the image to form an optical section.

The Swedish Ophthalmologist, 1**Allvar Gullstrand**, introduced slit illumination of the globe in **1911** to generate optical sections of its transparent tissues (Fig. 2) [1, 2].

However, this instrument did not emerge fully-formed; its evolution recruited contributions from a roll-call of great ophthalmologists of the period, still familiar names, today [3].

The formation of images by back-scatter from a thin ribbon of light traversing transparent tissue, requires a high-intensity, narrow slit of uniform illumination. Gullstrand’s original instrument used a Nernst lamp (a glowing ceramic rod), focused by a condenser lens onto the aperture of an adjustable slit and re-focused onto the eye by a further aspheric lens [1, 2].

**Fig. 3:**
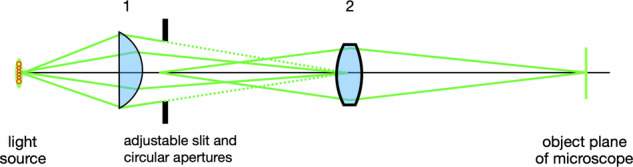
Köhler illumination to create a uniform field. The light source is focused by a convex lens (1) onto a second projecting lens (2); lens 2 focuses lens 1 onto the microscope’s object plane. In a slit-lamp microscope, adjustable slit and circular apertures are positioned immediately in front of lens 1 and it is these that are projected onto the eye.

By 1920, **Alfred Vogt** had substituted a “micro-arc lamp”, which was superseded by a glowing spiral filament in a nitrogen envelope. Köhler optics were required to erase the image of the filament from the microscope’s object plane (Fig. 3). Two lenses are used to project light onto the eye. The first focuses the filament onto the second lens, and adjustable slit and circular apertures in front of the first lens are focused by the second lens onto the eye [1, 4]. Vogt wrote an authoritative 3 volume textbook on the slit-lamp’s use and clinical findings.

**Leonhard Koeppe** is credited with incorporating the pre-existing binocular corneal microscope into the slit-lamp in 1920. Crucially, he introduced a common axis of rotation for the slit and microscope in 1926: an idea adopted in 1933 for the instrument of **Hans Goldmann** and **Wilhelm Comberg** [1].

**Fig. 4:**
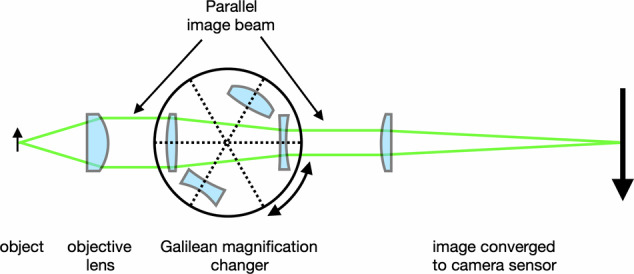
Littmann - Galilean magnification changer.

The crowning contribution was provided in 1950 when **Hans Littmann** introduced infinity-corrected optics, in which was placed a Galilean magnification changer: a ring of lens pairs which provide incremental variation in magnification (Fig. 4).

## Illumination techniques

### Wide field

**Fig. 5:**
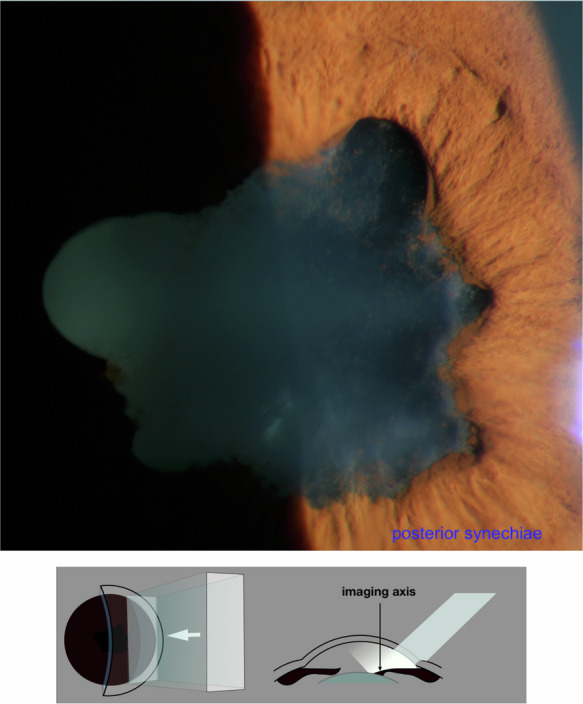
Anterior uveitis: posterior synechiae. The proximity of iris to lens is illustrated by shadow casting, using the edge of the collimated light field.

Contemporary ophthalmologists were slow to adopt Gullstrand’s slit illuminator. It was discrete from the imaging system; they already had access to binocular corneal microscopy and they could use the edge of a collimated light beam to demonstrate the relationships between anterior segment structures (Fig. 5).

Additional detail would have been available from specular reflections (Fig. 10) and shadow casting (Fig. 5). Fluorescein (see Fig. 19) had also been in use since its introduction by Paul Ehrlich in 1881 to demonstrate aqueous circulation in the rabbit [7].

### Optical sectioning

**Fig. 6:**
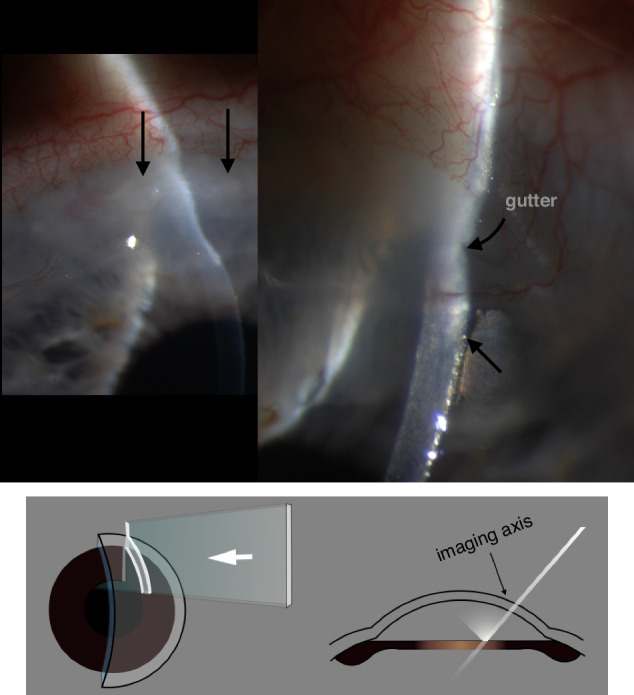
Slit images of a corneal melt: Granulomatosis with polyangiitis. The slit illustrates the changed corneal contours and characterises tissues according to transparency and the back-scatter of collimated light. Note the epithelial thickening and multifocal creamy stromal swelling and opacification, representing active melting (vertical arrows).The magnified image from the active apex of the gutter also shows more-central epithelial swelling and increased light scatter (lower arrow), suggesting oedema due, perhaps, to an overwhelmed endothelial pump. The gutter depth is less than 50% of corneal thickness and the anterior chamber is preserved.

**Fig. 7:**
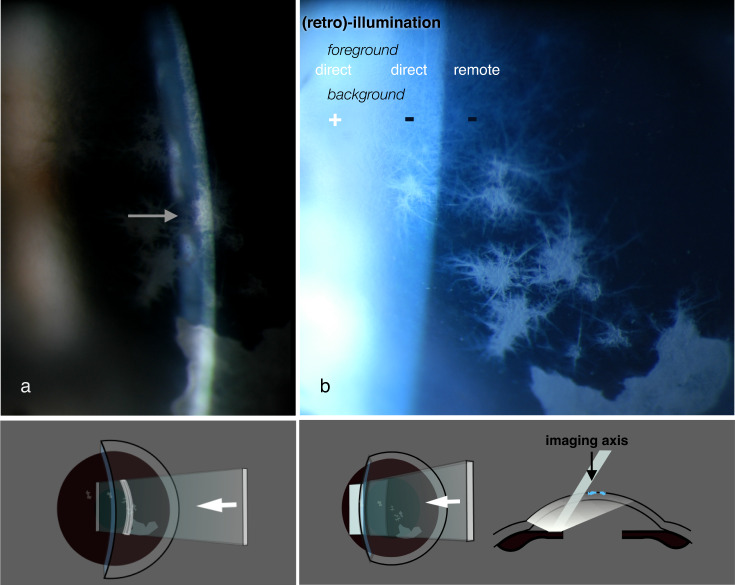
Infectious crystalline keratopathy (complicating penetrating keratoplasty). **a** Slit image: Arrow shows a shadow, cast by the epithelial pathology over the slit image of endothelium. **b** (Retro)-illumination: foreground / background combinations. Remote retro-illumination against a dark field enhances contrast and demonstrates pathological detail.

In the slit-lamp envisaged by Gullstrand, a bright ribbon of light slices through transparent tissues at an acute angle to the imaging axis. Histological features create refractive index changes which scatter light, returning some back towards the microscope. As the ribbon of light encounters progressively deeper layers of tissue, the images from back-scatter are spread in the meridian corresponding with the angle of illumination, so that the returned light forms an oblique optical section (Figs. 2, 6, 7a).

The finer the slit, the better resolved the optical section, and depth resolution is improved when the light source and imaging axis intersect in the tissue with greater obliquity. However, narrowing of the slit and increased obliquity reduce illumination intensity: a perpetual trade-off during slit-lamp microscopy.

### Retro-illumination

**Fig. 8:**
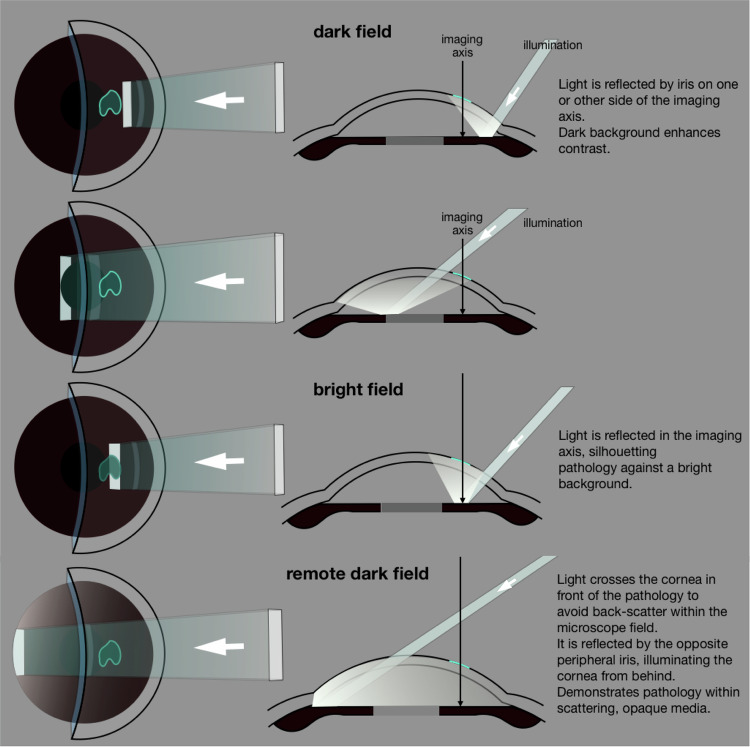
Slit configurations for dark-field, bright-field, and remote dark-field retro-illumination.

**Fig. 9:**
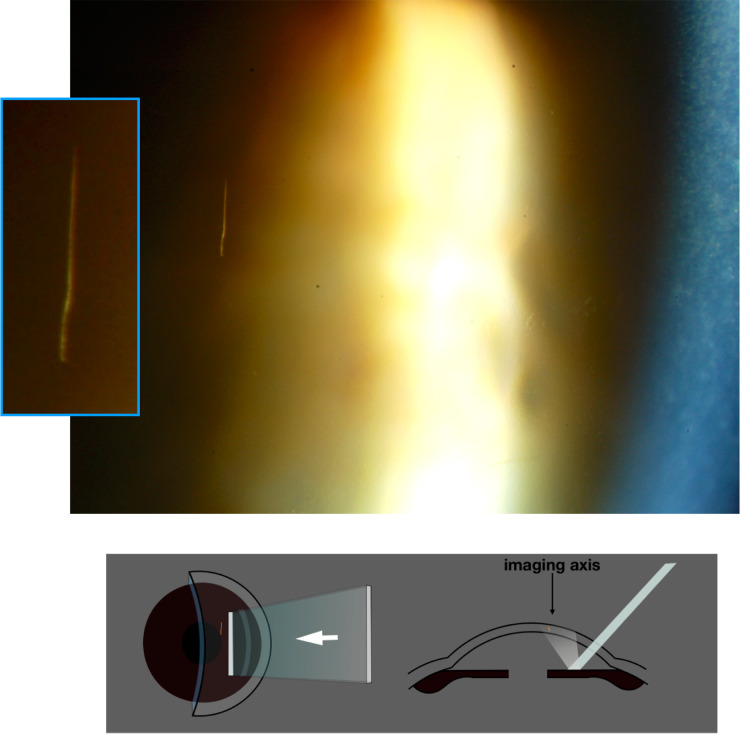
Tarantula hair in corneal stroma, demonstrated by retro-illumination. The slit has been angled, narrowed, and located on the iris so as to place the pathology at the junction between bright and dark fields.

When Goldmann configured the slit and microscope to rotate coaxially about the object plane, he may not have anticipated that its most powerful feature would become *deflection* of the slit *away* from their common axis. This enables light, reflected from more distant objects (usually the iris, during corneal microscopy), to illuminate the object plane from behind: retro-illumination (Figs. 7, 8 and 9).

Background brightness becomes crucial (ie: “*dark-field”* or “*bright-field*” retro-illumination) and slit-offset, width, and height are constantly re-adjusted to optimise this.

During clinical examination, a dark field provides high-contrast pathological detail; bright-field images may provide silhouettes of opaque or refractile objects, demonstrating topography in the x-y plane.

#### The most information-rich area of a slit image usually lies between the bright and dark background field

(Figs. 9, 10).

**Fig. 10:**
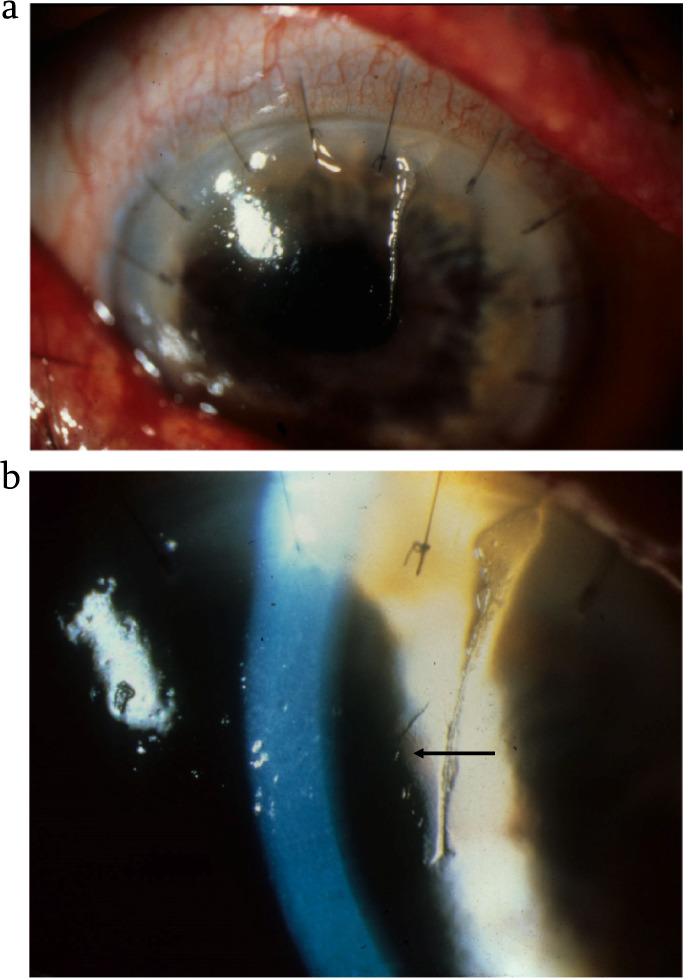
Mucus filament over a corneal graft. **a** Wide-field illumination - specular reflections show a mucus filament, anchored centrally and drawn upwards by the upper lid. **b** Retro-illumination with object placement between the bright and dark fields identifies the pathology to be a corneal epithelial defect (arrow).

#### Remote dark-field retro-illumination

Pathology can become obscured when light is scattered by the surrounding media; however, this may be overcome by *remote dark-field* retro-illumination. The light beam is directed through the air space in front of the cornea, to be reflected by peripheral iris in the opposite angle (see Fig. 8 and video).

#### Surface contours during retro-illumination

When light, reflected by deep structures, emerges through transparent tissues, it is refracted by their surface contours. A corneal depression acts as a concave lens and light is diverged, creating a bright ring with dark centre. Conversely, surface elevations act as convex lenses, which converge light, creating a central bright zone.

#### Meniscus retro-illumination; demonstration of tear cells

**Fig. 11:**
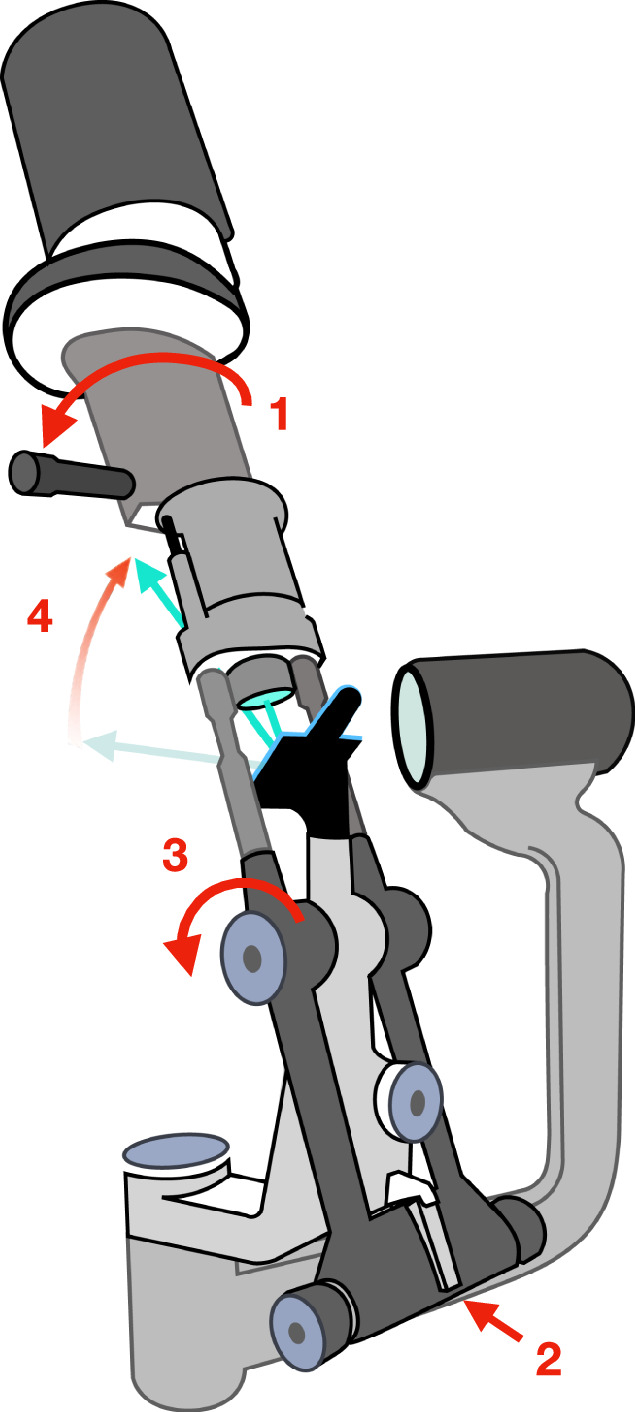
Haag Streit slit lamp, configured to provide an upward-sloping, horizontal ribbon of light for demonstration of cells in the superior tear film meniscus. **1** Rotate slit mask around the column axis, making its projected image horizontal. **2** Release lock on column. **3** Tilt the column forward: the mirror angle is fixed, therefore **4** the beam tips upwards.

**Fig. 12:**
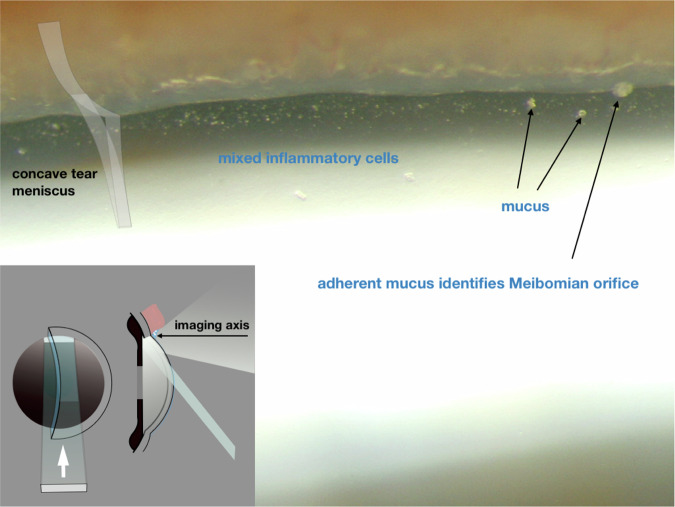
Tear cells and mucus complicating meibomianitis.

When an upward-sloping, horizontal ribbon of light, is reflected by peripheral iris and re-emerges from the eye, the superior tear meniscus presents a cylindrical, concave final surface. This gives a band of graded bright-to-dark-field retro-illumination, paralleling the lid margin, in which microscopy can demonstrate the cellular population of the tear film (Figs. 11 and 12).

The trans-illuminated cells in the tear film may act as point sources of light on account of the interference patterns which emerge from them [8].

For retro-illumination of the superior tear meniscus, the slit is configured for remote dark-field retro-illumination (Fig. 8); however, a horizontal beam is angled upwards to illuminate the superior peripheral iris, paralleling the upper lid (see Fig. 11).

### Specular reflection

The smooth, convex tear film acts as a convex spherical mirror, which reflects the light beam into the imaging system: the first Purkinje image^2^. Surface irregularities disturb this image. They act as tiny, inclined, convex or concave mirrors, which give rise to glistening fragmentations of this specular reflection (Figs. 6, 10). In this way, they can depict the architecture of a surface; particularly, the corneal endothelial mosaic.

#### Non-contact corneal endothelial specular microscopy

The intense, homogeneous reflection of the slit beam by the tear film (first Purkinje image) overwhelms the faint reflection from endothelium (second Purkinje image). Endothelial microscopy requires these to be separated, then the latter, selected.

**Fig. 13:**
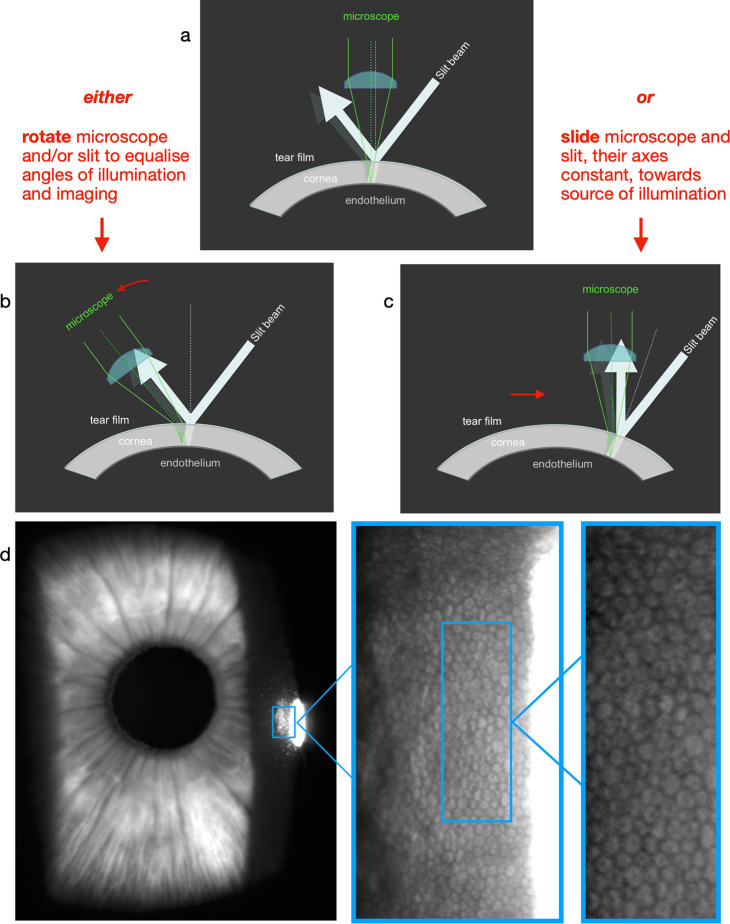
Non-contact corneal endothelial microscopy.

Conveniently, a single slit beam, approaching the cornea at an acute angle, generates epithelial and endothelial reflections, which are separated spatially (Fig. 13a). They can be displayed side by side in the microscope field by rotating the microscope and/or slit beam to equalise angles of incidence and reflection (Fig. 13b).

However, the slit’s appropriate angle of incidence is more reliably selected using corneal curvature: the angle of reflection becomes more acute as the microscope and slit are slid, angles constant, towards the light (Fig. 13c). The faint, speckled reflection, adjacent to the first Purkinje image, can then be expanded to demonstrate the endothelial mosaic (Fig. 13d).

## Access to remote structures

### Direct examination of the posterior segment ***(Lens and vitreous)***

All the principles of examination that apply in the cornea can be repeated as the focal plane is advanced through deeper transparent tissues. Retro-illumination demonstrates the anterior capsule of the crystalline lens against light returned from the concavity of the posterior capsule; the posterior capsule and vitreous are shown against the fundal red reflex.

By swinging the slit beam from one side to the other, the depth of vitreous pathology can be demonstrated by parallax with respect to the red reflex (see video “vitreous opacities in syphilis”).

**Fig. 14:**
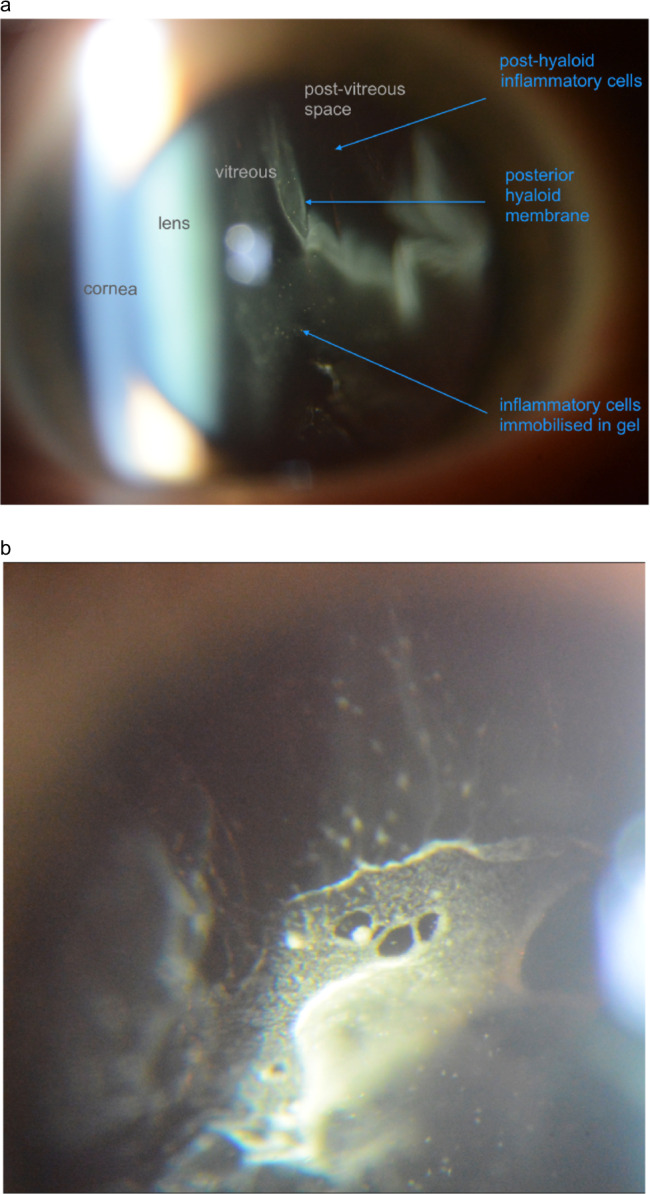
Examination of lens and vitreous (case of posterior uveitis). **a** slit image including cornea, lens, vitreous with immobilised cells, posterior hyaloid membrane, post-vitreous space. **b** cellular deposits on the posterior hyaloid membrane, rotated into the microscope field by a glimpse down and ahead.

Even when cells in the vitreous gel or post-vitreous space are too small to be resolved by the microscope, they can become visible by retro-illumination: the passage of light through a spherical object induces interference images, which makes it into a secondary light source (Figure 14a) [8].

Such vitreous cells may remain immobilised for long periods, historical and inactive. In contrast, cells suspended in, or precipitating from, the post-vitreous space indicate active disease. They can accumulate on the posterior hyaloid membrane as sheets or beads, analogous to keratic precipitates on corneal endothelium (Fig. 14b).

To image this directly, the inferior gel can briefly be lifted into the microscope field by asking the patient to glimpse down and ahead (Fig. 14b).

#### Expanding radial cords of vitreous cells suggest ocular lymphoma

**Fig. 15:**
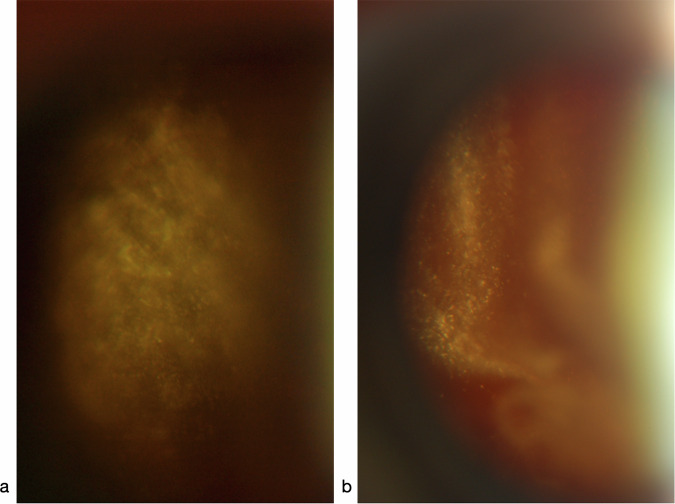
B-cell Non-Hodgkin’s lymphoma: expanding radial cords of vitreous cells. Vitreous contains radial cords of cells: **a** expanding centrally within the gel at presentation; **b** thinning centrally, 3 months after the start of treatment.

In B-cell lymphoma, lymphocytes may access the vitreous from its anterior periphery, forming radial cords which expand centrally (Fig. 15a): perhaps the result of continuing mitotic activity as they migrate through the gel. It is from the central gel that they are lost, and such cords thin centrally after treatment (Fig. 15b).

### Indirect examination of the posterior segment ***(posterior vitreous and retina)***

**Fig. 16:**
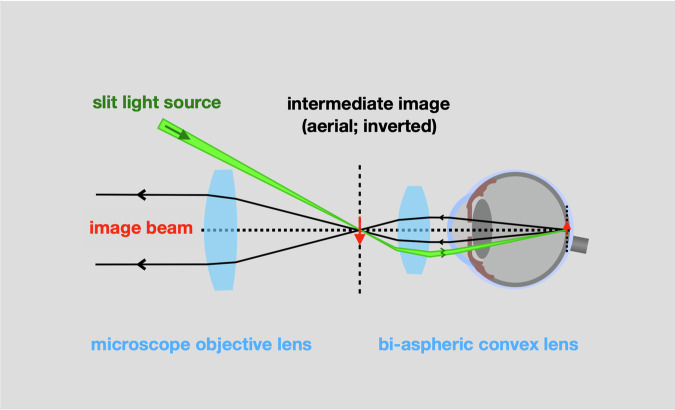
Optics of indirect retinal examination by slit-lamp microscopy.

A high-power, hand-held, bi-convex aspheric lens can create an intermediate, inverted aerial image of the retina or posterior vitreous (Fig. 16). Structures deep in the eye are re-presented in a plane in front of the aspheric lens; a plane that is accessible to slit-lamp microscopy and the various imaging techniques that have been discussed.

Just as the optics of the eye and bi-convex lens focus the retina onto this intermediate image plane, so the slit beam, falling on the aerial image, is projected back to illuminate the corresponding retina (Fig. 16). As the ophthalmologist illuminates and examines the intermediate image with the slit-lamp, he senses that he is interacting with the retina, itself.

### Mirror examination of the superior fornix

Structures in the superior fornix are difficult to examine using the horizontally-mounted slit-lamp microscope. When tissues are presented by single or double lid-eversion, the applied tension disorganises their anatomical relationships and interferes with blood flow.

**Fig. 17:**
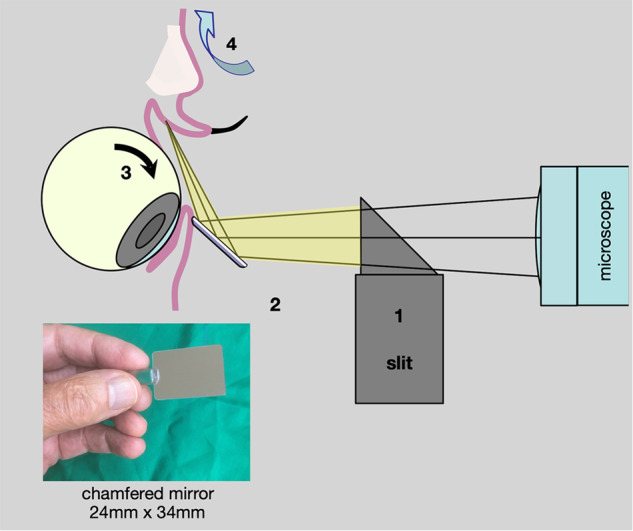
Mirror examination of the superior fornix. **1** Illuminate the lower lid **2** rest surface-silvered mirror against lower lid (45°) **3** patient looks down **4** retract upper lid (traction over brow or rotate cotton bud).

**Fig. 18:**
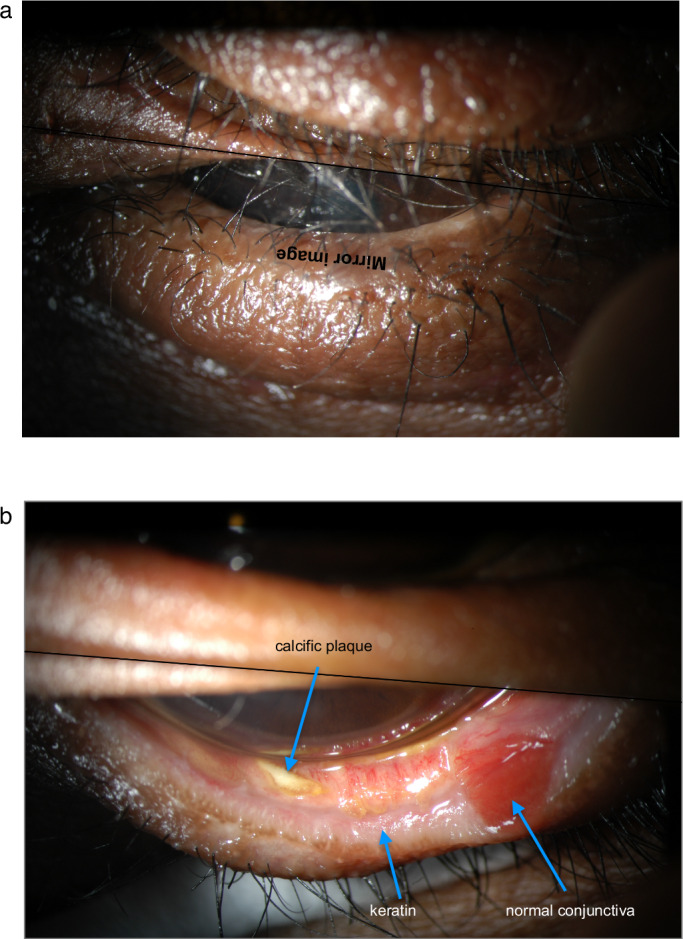
Mirror examination of the superior fornix. Stevens-Johnson Syndrome: investigation of a central corneal epithelial abrasion. **a** Inverted image of the upper lid margin demonstrates disorganised lashes with trichiasis. (Black line shows upper edge of mirror.) **b** Retraction of upper lid (rotated cotton bud) identifies a calcific plaque.

However, they can be observed, undisturbed, if a surface-silvered mirror is rested against the lower lid at 45°, the patient looks down, and the upper lid is gently retracted by traction over the brow or rotation of a cotton bud (Fig. 17). The precise field is selected by small angles of rotation and tilt of the mirror (Fig. 18).

## Selective imaging

### Wavelength restriction

A light source can be restricted, due to its indigenous spectrum or by filtration, to enhance or exclude the representation of particular tissues, pathologies or reagents.

Green light excludes long-wavelength illumination, reducing reflection by blood or retinal pigment epithelium, and this can be used to increase contrast during anterior and posterior segment vascular imaging. Haemoglobin video imaging (HVI) is an imaging modality which renders erythrocytes black by tailoring the illumination waveband to match a long-wavelength peak in the haemoglobin absorption spectrum (see below).

Wavelength restriction also increases resolution in optical systems by reducing chromatic aberration.

### Fluorescence

**Fig. 19:**
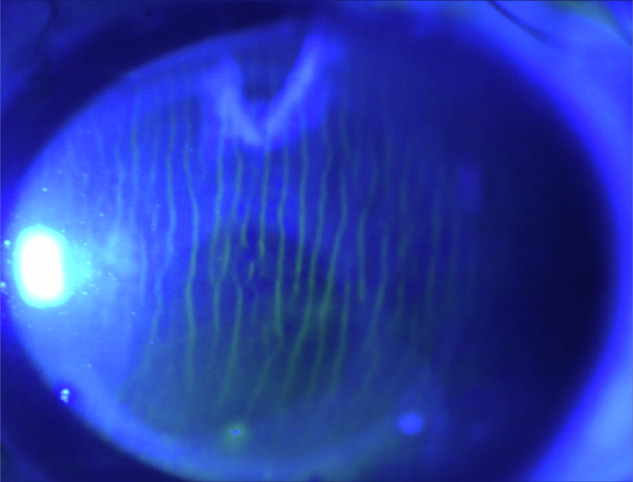
fluorescein depicts variations in tear film thickness. Intensity of fluorescence from a fluorescein-stained tear film varies according to its thickness. This can demonstrate characteristic vertical corneal folds in ocular hypotension (illustrated here); also, epithelial bullae with surrounding menisci in ocular hypertension.

Fluorophores are molecules which absorb light energy, re-emitting it at longer wavelength. For fluorescein, blue light excites longer-wavelength (green) fluorescence (excitation maximum 495 nm, emission maximum 520 nm). When illumination and observation are undertaken through “excitation” and “barrier” filters which do not overlap, the image is confined to fluorescence alone, and all non-fluorescent material is excluded. This most powerful method of selective imaging can precisely locate anatomical compartments or reagents. Topical fluorescein demonstrates the distribution of tears (hence menisci and corneal epithelial contours—see Fig. 19) and probes corneal epithelial damage or permeability; intravenous injection can reveal blood flow and vascular competence [9], and the circulation of aqueous [7].

The relationship between fluorescein concentration and fluorescence is non-linear. Its absorption and emission spectra overlap and, at high concentration, detectable fluorescence is suppressed due to its absorption by surrounding fluorescein molecules. Such “quenching” is exploited during the Seidal test, in which leaking aqueous is localised by its focal enhancement of fluorescence where it *dilutes* a concentrated fluorescein solution.

#### Vertical corneal folds signify ocular hypotension

## Slit-lamp video-microscopy

### Haemoglobin video imaging

Tissue selection by wavelength restriction usefully identifies erythrocytes in the conjunctival and episcleral circulations, enabling video recording of blood flow through microcirculations with single cell (erythrocyte) resolution [10, 11].

**Fig. 20:**
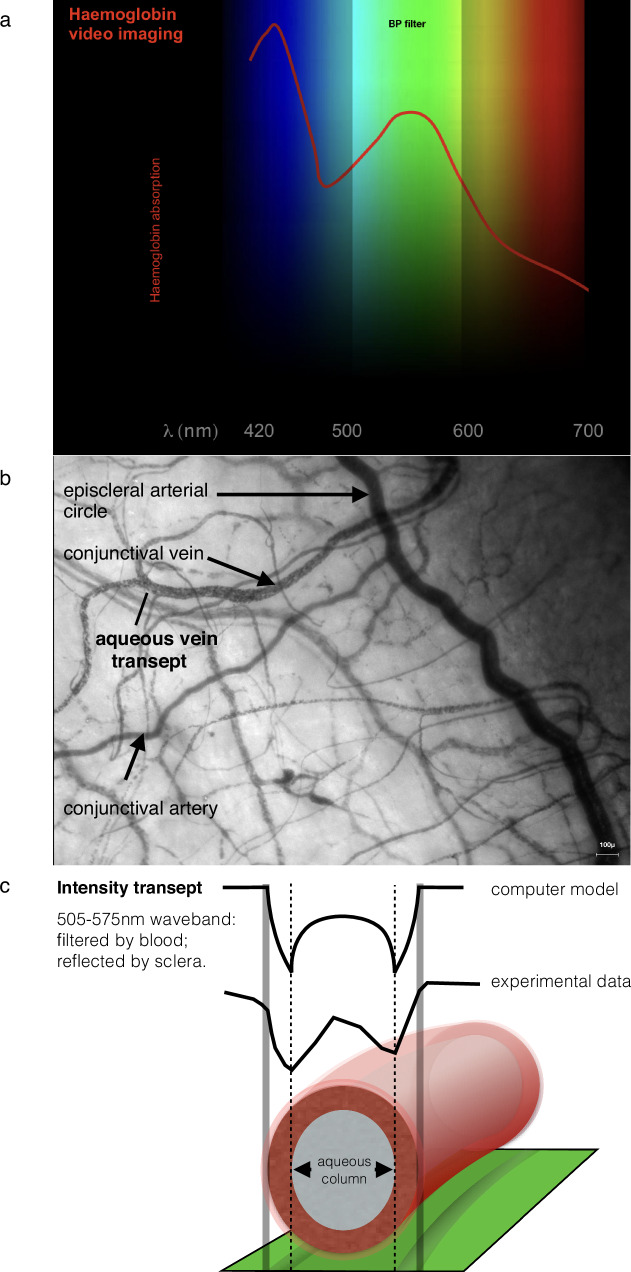
Haemoglobin video imaging and aqueous column cross-section area.

Illumination of the microcirculation is restricted to a waveband corresponding with the long-wavelength peak in the haemoglobin absorption spectrum (Fig. 20a). This waveband is absorbed by erythrocytes before and after its reflection by sclera, therefore blood vessels appear black against a bright background.

#### Haemoglobin Video Imaging and aqueous column cross-section area

When light is transmitted through aqueous veins, transepts of the image have a characteristic profile and the diameter of the aqueous column is defined by separation of the intensity minima (Fig. 20b, c). Aqueous column cross-section area can be used as a surrogate for aqueous flow volume [12], enabling studies on aqueous outflow and glaucoma management [13, 14].

Other clinical applications of haemoglobin video imaging have included studies of microvascular anatomy and physiology [10, 11], the consequences of VEGF inhibition on microcirculations [15] and the diagnosis of critically-elevated orbital tension in patients with thyroid eye disease, by observation of reversed conjunctival venous drainage [16].

#### Autofocus for video-microscopy

Virtually all modern slit-lamps employ parallel light optics and a Littmann - Galilean magnification changer. This delivers a parallel image beam, which can be converged to a video camera (Fig. 4). However, histological examination or characterisation of blood flow require images to remain perfectly stable in the X-Y image plane and the Z-axis (autofocus). Constant magnification is also required.

X-Y stabilisation can be undertaken either during or after a video recording, but refocusing must keep pace with image acquisition, and documentation of erythrocyte flow demands high video frame-rates.

Autofocus for video is normally “active”, meaning that the object plane is continuously located by measuring the delay encountered during reflection of pulses of energy (usually ultrasound or infra-red radiation). The focusing and imaging modalities must differ and the object must lie at, or at a constant distance from, a reflective surface. This is unsuitable for microcirculations, which undulate through transparent media.

Alternatively, during “passive” autofocus, location of the object plane is not considered and focusing is adjusted purely to optimise image quality. Sharpness (contrast) in the image is quantified, before and after a random change in focusing power: if it improves, the change is increased incrementally until sharpness begins to decline. A deterioration in sharpness prompts reversal of the focusing change. Needless to say, any refocusing system which intermittently deteriorates image sharpness is incompatible with video.

These limitations have prompted the development of autofocus systems which can determine the required direction of refocusing using the image, itself. In one such method (“phase detection”), two images of the object converge to cross at a single sensor. Whether they cross beyond or before the sensor determines if more or less optical power is required. When they cross at the sensor, the image is considered to be focused. However, this method requires bespoke lenses, sensors and software.

#### Autofocus by contrast analysis of an inclined image plane

Therefore, we have developed a new autofocus method for video-microscopy, in which the image plane is established by contrast analysis of an inclined image. This can continuously determine both the required direction and power of refocusing. An adjustable focus air-lens is used for refocusing, minimising variations in magnification and resolution [17], and the device is compatible with any infinity-corrected microscope (paper in preparation). We can now maintain stable focus during haemoglobin video imaging studies, keeping pace with video rates of 60 frames per second (see video “autofocus by contrast analysis of an inclined image plane”).

This technique enables microvascular blood flow to be sampled in the conjunctiva and episclera for long periods, without the need for a skilled operator. It is being used to investigate normal microvascular physiology and aqueous outflow, and to monitor pathological perfusion in ocular and systemic diseases.

## Conclusion: optical examination of the eye

The slit-lamp has enabled the microscopist to dissect and display the eye using, not a scalpel, but a light beam. During clinical slit-lamp microscopy, many optical techniques are available to picture the eye’s anatomy, function and pathology.

Retro-illumination presents pathology in the x-y plane by obscuration or by forward scatter of reflected light; optical sectioning locates that pathology in the z-axis and depicts it by back-scatter (Figs. 6–9). The two approaches produce very different images; they are complementary, and they can be combined in a single examination.

The ophthalmologist unclamps the slit-beam, dissociating it from the imaging axis, and he controls its angle throughout the examination. Transverse sweeps are performed, with a broad, then a fine, slit. He observes the bright and dark retro-illumination fields (particularly, the band at which they meet) and scrutinises the optical sections he creates, compiling an intellectual 3-dimensional image.

Any object plane that is too deep to be examined directly can be lifted out of the eye, using a hand-held bi-convex aspheric lens, to form an inverted aerial image, accessible for interrogation by all the optical methods employed in the anterior segment.

The superior fornix can be examined using a mirror.

Through the 20th Century, the slit-lamp microscope has enabled the imaging of living connective tissues, epithelia, nerves, microcirculations and cells of the immune system, often with single-cell resolution. It has transformed our understanding and management of ophthalmic disease. The addition of 21st Century technology holds the prospect that pathophysiology, sampled in the eye, will facilitate the differential diagnosis and management of systemic diseases.

## Supplementary information

Remote dark-field retro-illumination/Parallax/autofocus by contrast analysis of an inclined image plane (Conjunctival and episcleral microcirculations).

## Footnotes

1 *Gullstrand is the only ophthalmologist to date to receive a Nobel Prize for “Physiology or Medicine”. This celebrated, in 1911, his studies on the dioptrics of the eye and accommodation* [2, 5]*. He later became chair of the Nobel committee, and is notorious for a report, arguing against Einstein receiving a prize for the Theory of General Relativity, which he considered too speculative* [2, 5, 6].

2 *The 19th century physicians Jan Evangelista Purkinje and Louis Sanson observed that, when illuminated, the eye reflects 4 separate images of the light source. These represent reflections from each major refractive interface* [4]*:*

1. *air - cornea (tear film and corneal epithelium),*
2. *cornea (endothelium) - aqueous,*
3. *aqueous - lens (anterior lens capsule)*
4. *lens (posterior capsule) - vitreous: concave surface, therefore inverted.*
